# Impact of indoor air pollution from the use of solid fuels on the incidence of life threatening respiratory illnesses in children in India

**DOI:** 10.1186/s12889-015-1631-7

**Published:** 2015-03-28

**Authors:** Ashish Kumar Upadhyay, Abhishek Singh, Kaushalendra Kumar, Ashish Singh

**Affiliations:** International Institute for Population Sciences, Govandi Station Road, Deonar Mumbai, 400 088 India; Indian Institute of Technology Bombay, Mumbai, India

**Keywords:** Indoor air pollution, Life threatening respiratory illnesses, Young Lives Study, Panel data, Two-stage random effects regression, India

## Abstract

**Background:**

India contributes 24% of the global annual child deaths due to acute respiratory infections (ARIs). According to WHO, nearly 50% of the deaths among children due to ARIs is because of indoor air pollution (IAP). There is insufficient evidence on the relationship between IAP from the use of solid fuels and incidence of life threatening respiratory illnesses (LTRI) in children in India.

**Methods:**

Panel data of children born during 2001–02, from the Young Lives Study (YLS) conducted in India during 2002 and 2006–07 was used to estimate the impact of household use of solid fuels for cooking on LTRI in children. Multivariable two-stage random effects logistic regression model was used to estimate the odds of suffering from LTRI among children from households using solid fuels relative to children from households using other fuels (Gas/Electricity/Kerosene).

**Results:**

Bivariate results indicate that the probability of an episode of LTRI was considerably higher among children from households using solid fuels for cooking (18%) than among children from households using other fuels (10%). Moreover, children from households using solid fuels in both the rounds of YLS were more likely to suffer from one or more than one episode of LTRI compared to children from households using solid fuels in only one round. Two-stage random effects logistic regression result shows that children from households using solid fuels were 1.78 (95% CI: 1.05-2.99) times as likely to suffer from LTRI as those from households using other fuels.

**Conclusion:**

The findings of this paper provide conclusive evidence on the harmful effects of the use of solid fuels for cooking on LTRI in India. The Government of India must make people aware about the health risks associated with the use of solid fuels for cooking and strive to promote the use of cleaner fuels.

## Background

Acute respiratory infections (ARIs) kill 0.94 million children under five years of age annually [1]. The burden of ARIs in developing countries is considerably higher than that in developed countries [2]. In India in 2010, 24% of the total deaths among children under five was due to ARIs [3]. In terms of incidence, 151 million new ARI cases occur annually among children under five in developing countries [4-6]. Many of these result in the death of young children. For example, in India, about 0.16 million male children and 0.21 million female children below the age of five died due to ARIs in 2005 [7]. Recent estimates suggest that about 0.41 million young children died of ARIs in India in 2010 [3].

Research on the subject indicates that indoor air pollution (IAP) from the use of solid fuels for cooking/heating is one of the important risk factors of ARIs [7-11]. Exposure is particularly high among women and young children, who spend most of their time near the domestic hearth [12]. According to WHO [1], nearly half of the deaths among children due to ARIs is because of IAP. Recent data from India suggests that the use of solid fuels was responsible for 20% of the total deaths among children in the age group, 1–4 years [13]. According to IIPS & ORC Macro [14], only 4% of the children living in households using electricity/LPG had ARI symptoms compared to 7% of the children belonging to households using animal dung as fuel [14].

An important factor while examining the role of IAP in causing health risks is the permeability or ventilation in the dwelling [15-18]. A study carried out by Dasgupta, Huq [17] in Bangladesh showed that ventilation such as roof and wall permeability reduced the average household pollution level greatly. Akunne, Louis [15] also found an association between permeability and impact of IAP. On the other hand, Pitt, Rosenzweig [19] concluded that improving ventilation by increasing the permeability of roofs and walls had no significant effect on health. The study by Gajate-Garrido [20] could not establish the benefits of higher permeability in the dwelling. The issue of the use of hazardous fuel and permeability of dwelling is particularly important in India because, of the 247 million Indian households, about 173 million use solid fuels such as firewood, crop residual, cow dung and cake coal/charcoal. Of these 173 million households, 75 million do not have a separate kitchen [21].

A number of Indian studies have reported an association between IAP caused by cooking fuel and risk of ARIs among children under the age of five [8,10,11,22-27]. Notably, majority of these studies are based on cross-sectional data, and hence these studies fail to develop any causal relationship between IAP caused by cooking fuel and risk of ARIs. None of the Indian studies have included roof and wall permeability in the analysis. Moreover, the impact of IAP on ARIs is also likely to depend on the number of other women (like aunt, grandmothers, etc.) present in the household. Pitt, Rosenzweig [19] argued that the presence of other women in the household is likely to reduce young children’s exposure to IAP. Mothers with young children are likely to spend less time close to the stove if other women like grandmothers or aunts are present in the house [19]. It is important to mention that none of the Indian studies have included the presence of other women in their statistical models.

Our study complements and augments existing literature by examining the impact of IAP from the use of solid fuels for cooking on the incidence of life-threatening respiratory illnesses (LTRI) using panel data. The panel structure of the data allows our analysis to capture the dynamic nature of the household and community level variables, isolate the effect of omitted variables, reduce collinearity among exposure variables and provide robust causal effect of exposure variable on the outcome variable. We also account for the permeability of the dwelling and the presence of other women in the house while examining the impact of use of solid fuels for cooking on the incidence of LTRI.

### Data and methods

#### Data

We use data from the first and second rounds of the Young Lives Study (YLS), which was conducted in the state of Andhra Pradesh in India during 2002 and 2006–07. Young Lives is an international longitudinal study investigating the changing nature of childhood poverty. About 12000 children are being followed in four countries: Ethiopia, Peru, Vietnam and India (Andhra Pradesh). Each country has two cohorts: younger cohort and older cohort to be followed over a period of 15 years. The younger cohort consists of about 2000 children born in 2001–2002 and the older cohort consists of about 1000 children born in 1994–1995 [28,29]. The YLS is conducted every three/four years to collect data on a range of indicators related to the growth and development of children [28-30].

A multistage sampling design was adopted in YLS. In the first stage, two districts were selected from each of the three geographic regions (Coastal, Rayalseema and Telangana) of the state of Andhra Pradesh. In the second stage, 19 (15 from rural areas and 4 from urban areas) sentinel sites (administrative blocks or ‘mandals’) were selected from the six selected districts. In addition, one sentinel site was selected from the urban slums of the city of Hyderabad. In the third stage, villages were selected from rural sentinel sites and wards were selected from urban sites. All the households with a one year old child (born in 2001–2002) or an eight year old child (born in 1994–95) in the selected villages and wards were included in YLS. Overall, 2011 households (with 2011 children) in the younger cohort (born in 2001–2002) and 1008 households (with 1008 children) in the older cohort (born in 1994–95) were included in the first round of YLS, which was conducted in 2002 (for details of YLS sampling design, see [6,28,29]. As the objective of this study is to analyze the impact of the use of solid fuels on the incidence of LTRI in children under six years, we include only the younger cohort (born in 2001–02) in the analysis. This is again a reason for using only the first two rounds of YLS that is, 2002 and 2006–07 in the analysis.

The second round took place between late 2006 and early 2007 and included 1950 children in the younger cohort. The attrition rate between the two rounds was about 3% [31]. While the pooled analysis presented in this paper is based on observations on 3961 children, the panel analysis is based on 1950 children.

### Outcome variable

The outcome variable of interest is the incidence of LTRI. Both rounds of YLS asked mothers two questions related to life threatening illnesses:

1. In the (reference period), has the child had any serious illnesses or injuries when you really thought he/she might die? (Yes/No/Don’t Know)
2. What were the illnesses or injuries?

If the mother reported pneumonia, severe cough, asthma, acute respiratory problems and high fever in response to the second question, then we coded LTRI as ‘1’ and otherwise‘0’. Hence, LTRI is a binary indicator variable, which takes value ‘0’ when no episode of LTRI occurred and ‘1’ otherwise. By using LTRI instead of minor illnesses, we were able to exclude seasonal health problems in our analysis.

### Independent variable

The independent variable of interest in the present study is the presence of indoor air pollution from the use of solid fuel for cooking. The survey gathered information on the main type of fuel used for cooking. Cooking fuels like wood, charcoal, coal and cow dung were coded as solid fuels. Electricity, gas and kerosene were coded as other cooking fuels (or cleaner fuels). The United States Environmental Protection Agency’s Standard for the 24-hour average of PM_10_ is 150 ug/m3 [32]. Since kerosene has emission levels (PM_10_ 134 ug/m^3^) below the recommended standard [17], we included kerosene in the category of ‘other cooking fuel’.

### Other key variables

The other key variables included age of the child (in months), sex of the child (female; male), wall permeability (non-permeable; permeable), roof permeability (non-permeable; permeable), child’s nutritional status (Height-for-age z-score > = − 2SD; Height-for-age z-score < −2SD), wealth index (poorest; poorer; middle; richer; richest), presence of other women at home (no/yes), household crowding (<3 persons per room; > = 3 persons per room), and the interactions of cooking fuel with wall permeability, roof permeability and sex of the child.

If the wall of the house was made of matting, wood/branches, cement bag, fibreboard/chipboard or stone, it was classified as permeable. Walls made of any other material were classified as non-permeable. If the roof was constructed of straw/thatch, tiles/slates, wood/plank or galvanised iron, it was classified as permeable. Roofs made of other materials were classified as non-permeable.

The child’s nutritional status was measured using height-for-age z-score. Children whose height-for-age z-score was below minus two standard deviations from the median of the reference population were considered short for their age or stunted. Such children are also considered chronically malnourished [14].

We also generated a wealth index based on household assets (including radio, refrigerator, bicycle, television, motorbike/scooter, car, pump, sewing machine, mobile, phone, landline telephone, fan, almirah, clock, table, chair, sofa, bedsheet and animals), household quality (including wall, roof and floor) and services (including electricity, drinking water, toilet facility) using principle components analysis. The generated index was then coded into five categories. Based on the wealth index, the lowest 20% of the households were coded as the poorest, the next 20% as poorer, and so on.

A number of other socioeconomic, demographic and residence related variables affect the health of children [11]. Accordingly, we controlled for number of siblings below five years of age, mother’s schooling (0–4 years; 5–9 years; 9+ years), mother’s working status (not working; agricultural work; other work), schooling of household head (0–4 years; 5–9 years; 9+ years), religion of household head (Hindu; Muslim; others), caste of household head (Scheduled Caste/Scheduled Tribes; other backward caste; others), household’s size, income shocks (no/yes), residence (rural/urban), exposure to outdoor air pollution (no/yes), percentage of literate mothers in the community, and ecological zone (others/inland plane) as control variables in the statistical models.

Income shocks refer to the loss of job or source of income that significantly decreased the economic welfare of the household. Income shocks at the household level were assessed by the answers to the following question asked in the two rounds of YLS:

‘In the last four years has the household suffered loss of job/source of income/family enterprise? (Yes/No)’.

The respondents were asked to report the ecological zone to which they belonged. The ecological zone is a pre-coded variable with four categories (inland plane, coastal plane, rain forest, and hill). Since 78 of the 98 communities included in YLS belonged to the ‘inland plane’, we coded the ecological zone into two categories (inland plane and others). Direct questions were asked in YLS to assess the exposure of the communities to outdoor air pollution from garbage burning, industrial activity and transportation. This information was used to create the variable, ‘exposure to outdoor air pollution’. If the community was exposed to any of the three afore-mentioned sources, ‘exposure to outdoor air pollution’ was coded as ‘1’ and otherwise, ‘0’.

## Methods

The association between outcome variable and exposure variables were examined using pooled and panel data estimation. For pooled data estimation, we pooled the data from the first and second rounds of YLS. This increased the sample size, thereby giving a more precise estimate of the relationship between exposure variables and the outcome variable. An obvious problem with the pooled data analysis is that it is useful only when the relationship between exposure variables and outcome variable remains constant over time [6,29,33]. However, this is rarely the case. Therefore, we used panel data estimation to capture the dynamic nature of the relationship between the independent variable and the outcome variable. We treated the two rounds of YLS as two panels. By virtue of capturing the dynamic nature of the relationship, the panel data estimates are more precise, and have greater power [9,33].

We have used a multivariable two-stage random effects logistic regression model to assess the relationship between the independent variable and the outcome variable. The multivariable two-stage random effects model was used to account for the potential endogeneity while estimating the impact of children’s nutritional status on LTRI. On the one hand, children’s poor nutritional status might increase the risk of LTRI [30]. Besides, repeated incidence of LTRI might retard the growth of children during the early years [34,35]. We used mother’s height as an instrument. Mother’s height is highly correlated with children’s nutritional status during the early years [36,37] but is independent of the incidence of LTRI among children. The technical details of the two-stage random effects logistic regression model are given in Appendix. All the other key variables were tested for multi-collinearity before being included in the regression models. All the statistical computations were done in STATA 12.0. All the standard errors were clustered at the community level.

### Ethics approval

Our study is based on a secondary dataset with no identifiable information on the survey participants. This dataset is available in public domain for research use and hence no approval was required from any institutional review board. The data can be downloaded from the website of the United Kingdom Data Archives University of Essex after taking permission. The data for the current study was downloaded from the afore-mentioned website after taking permission (I.D. No. 70895).

## Results

Table 1 presents the percentage of child/household and community characteristics according to the type of cooking fuel for Round-1 (2002), Round-2 (2006–07) and the pooled data. In the first round, 75% of the households reported using solid fuels for cooking. It compares with 73% in the second round. The probability of LTRI remained almost unchanged from round-1 (16.6%) to round-2 (15.9%). In both the rounds, the probability of LTRI varied considerably by the type of cooking fuel. In round-1, 18% of the children from households using solid fuels were suffering from LTRI. In comparison, only 11% of the children from households using cleaner fuels suffered from LTRI. However, in round-2, children from households using solid fuels were twice as vulnerable (18% versus 9%) to LTRI as those from households using cleaner fuels. Children from households using solid fuels were twice as likely as children from households using cleaner fuels to be malnourished (round-1: 31% versus 16%; round-2: 42% versus 21%).

**Table 1:** **Percentage of child/household characteristics and community characteristics according to use of cooking fuel in round 1, round 2 and pooled data**

	**Round 1**			**Round 2**			**Pooled**		
	**Total**	**Cleaner**	**Solid**	**Total**	**Cleaner**	**Solid**	**Total**	**Cleaner**	**Solid**
**Child/household characteristics**									
Prob. of serious respiratory related illnesses	16.6	10.9	18.4	15.9	9.0	18.5	16.2	9.9	18.5
Prob. of being malnourished	27.2	16.3	30.8	36.1	21.2	41.7	31.6	18.8	36.1
Prob. of lowest birth weight quarter	44.0	35.6	49.9						
Boy	53.8	52.8	54.1	53.3	52.6	53.5	53.5	52.7	53.8
Age of child (in months)	11.8	11.7	11.9	64.3	64.1	64.3	37.6	38.9	37.2
**Mother's schooling**									
0-4 years	62.2	23.4	74.9	61.6	24.0	75.5	61.9	23.7	75.3
5-9 years	22.4	32.1	19.2	21.9	30.5	18.6	22.1	31.3	18.9
9+ years	15.5	44.6	5.9	16.6	45.5	5.7	16.0	45.1	5.8
**Wealth index**									
Poorest	20.2	0.8	26.6	20.0	0.4	27.4	20.1	0.6	27.0
Poor	19.8	3.6	25.1	20.1	2.4	26.7	19.9	3.0	25.9
Middle	20.1	6.9	24.5	20.0	5.2	25.5	20.1	6.0	25.0
Rich	19.8	23.0	18.8	20.0	28.3	16.9	19.9	25.7	17.9
Richest	20.0	65.7	5.0	20.0	63.7	3.5	20.0	64.7	4.3
**Schooling of household’s head**									
0-4 years	60.6	26.4	71.8	52.5	19.7	64.8	56.6	23.0	68.4
5-9 years	19.3	27.0	16.8	23.9	24.6	23.6	21.6	25.8	20.1
9+ years	20.1	46.6	11.5	23.7	55.6	11.7	21.9	51.3	11.6
Other women in the household	52.7	46.0	54.9	66.6	60.3	69.0	59.6	53.4	61.7
Wall permeability	19.9	5.0	24.8	12.4	7.1	14.4	16.2	6.1	19.8
Roof permeability	56.3	41.5	61.2	46.7	39.0	49.6	51.6	40.2	55.6
Prob. of income shock	5.1	2.8	5.8	1.2	1.7	1.0	3.2	2.2	3.5
**Crowding**									
<3 persons per room	32.5	47.6	27.6	49.3	68.5	42.0	40.8	58.5	34.6
> = 3 persons per room	67.5	52.4	72.4	50.7	31.5	58.0	59.2	41.5	65.4
**Community characteristics**									
**Place of Residence**									
Rural	74.9	22.2	92.2	74.4	24.5	93.2	74.7	23.4	7.3
Urban	25.1	77.8	7.9	25.6	75.5	6.8	25.4	76.6	92.7
Inland plane	60.8	57.5	61.9	58.3	52.4	60.5	59.6	54.9	61.2
% of households in the community (excluding the child’s household) using solid fuel	75.0	32.5	89.4	72.6	33.0	87.5	74.0	32.8	88.5
Community female education level	38.4	60.3	31.2	50.2	61.8	45.8	44.2	61.1	38.3
Outdoor air pollution	10.0	19.4	6.8	23.0	47.4	13.8	16.3	33.9	10.2
**Number of observations**	**2011**	**496 (25%)**	**1515 (75%)**	**1950**	**534 (27%)**	**1416 (73%)**	**3961**	**1030 (26%)**	**2931 (74%)**

Mother’s schooling, schooling of the household head and wealth index were much better in households using cleaner fuels than in households using solid fuels. Interestingly, households using solid fuels were more likely to have permeable walls and roof compared to households using cleaner fuels. Other women (like grandmother/aunts) were more likely to be present in households using solid fuels compared with households using cleaner fuels. Income shocks were more likely to be reported in households using solid fuels. Interestingly, in both rounds, the average level of female education was higher in those neighbourhoods where a higher percentage of households used cleaner fuels. Outdoor air pollution was more common in neighbourhoods surrounding households that reported using cleaner fuels.

**Figure 1:**
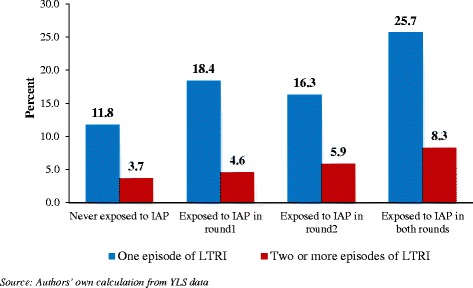
**Episodes of life threatening respiratory illnesses (LTRI) by exposure to indoor air pollution (IAP) from the use of solid fuels in different rounds of YLS.**

Figure 1 presents the episodes of LTRI by exposure to indoor air pollution caused by cooking fuel. The probability of at least one episode of LTRI in children from households using cleaner fuels in both the two rounds was 12% and 4%, respectively, while it was 26% and 8% respectively in children from households using solid fuels in both the rounds. The probability of one or two episodes of LTRI was considerably higher in children from households using solid fuels in both the rounds than in children from households using solid fuels in only one round.

Results of the multivariable two-stage random effects logistic regression model are shown in Table 2. The use of solid fuels was statistically associated with LTRI in pooled data regression, independent of the child’s age, gender, nutritional status, mother’s education, household wealth and other factors. Children from households using solid fuels were 1.71 (95% CI: 1.10-2.68) times as likely as children from households using cleaner fuels to suffer from LTRI. Wealth index was also statistically associated with LTRI. That is, an increase in wealth was statistically associated with decline in the risk of LTRI. Interestingly, the interaction term between cooking fuel and roof permeability was significant - indicating that the effect of solid fuels on the incidence of LTRI depended on the permeability of the roof. The use of solid fuels in houses with permeable roofs was associated with lower risk of LTRI in children (Odds ratio – 0.62; 95% CI: 0.41-0.93).

**Table 2:** **Multivariable two-stage random effects logistic regression model results for the impact of indoor air pollution on the incidence of Life Threatening Respiratory Illnesses among children under six years of age**

	**Life threatening respiratory illnesses**					
	**Pooled**			**Panel**		
**Exposure variable**	**OR (SE)**	**95% CI**	**p-value**	**OR (SE)**	**95% CI**	**p-value**
**Cooking fuel**						
Cleaner®						
Solid	1.71(0.39)*	(1.10-2.68)	0.018	1.78(0.47)*	(1.05-2.99)	0.031
**Gender**						
Female®						
Male	1.17(0.16)	(0.89-1.53)	0.257	1.18(0.28)	(0.75-1.87)	0.473
**Age of child (in months)**	0.99 (0.00)	(0.99-1.01)	0.814	0.99 (0.00)	(0.99-1.00)	0.757
**Stunting**						
HAZ > = − 2SD®						
HAZ < −2SD	1.07(0.17)	(0.78-1.47)	0.672	1.07(0.13)	(0.79-1.45)	0.555
**Wealth index**						
Poorest®						
Poorer	0.95(0.12)	(0.73-1.23)	0.695	0.95(0.13)	(0.73-1.24)	0.711
Middle	0.66(0.11)*	(0.48-0.91)	0.012	0.65(0.10)*	(0.48-0.88)	0.005
Richer	0.59(0.11)*	(0.40-0.86)	0.006	0.57(0.11)*	(0.40-0.82)	0.003
Richest	0.54(0.16)*	(0.30-0.97)	0.038	0.53(0.14)*	(0.32-0.88)	0.015
**Wall permeability**						
Non permeable®						
Permeable	1.06(0.58)	(0.37-3.10)	0.908	1.07(0.47)	(0.46-2.51)	0.873
**Roof permeability**						
Non permeable®						
Permeable	1.34(0.28)	(0.89-2.00)	0.160	1.35(0.31)	(0.86-2.12)	0.195
**Other women in the household**						
No®						
Yes	1.14(0.11)	(0.93-1.38)	0.204	1.15(0.13)	(0.92-143)	0.235
**Household crowding**						
<3 persons per room®						
> = 3 persons per room	1.14(0.13)	(0.90-1.44)	0.253	1.18(0.13)	(0.95-1.46)	0.126
**Fuel x gender**	0.93(0.14)	(0.69-1.25)	0.615	0.91(0.23)	(0.56-1.50)	0.718
**Fuel x wall permeability**	0.93(0.53)	(0.39-2.23)	0.902	0.92(0.42)	(0.38-2.24)	0.855
**Fuel x roof permeability**	0.62(0.13)*	(0.41-0.93)	0.022	0.60(0.16)*	(0.36-0.99)	0.049
**Other controls**	Yes			Yes		
**Observation**	3961			1950		

Note: Odds ratios presented in the table are adjusted odds ratios. Standard errors are in parentheses, * p < 0.05.

Other controls: age of child, mother’s schooling, schooling of household head, household size, no. of children below age 5, religion, caste, income shock, place of residence, outdoor air pollution, ecological zone, community female education level.

Use of solid fuels was also statistically associated with LTRI in panel regression (Table 2). The odds ratio was of the order of 1.78 (95% CI: 1.05-2.99). Wealth index was also associated with LTRI. The interaction between cooking fuel and roof permeability was statistically significant in panel data regression. Notably the effects became stronger when panel data regression was used.

The presence of other women (like grandmother/aunts) and household crowding were not statistically associated with the incidence of LTRI. The interaction between cooking fuel and gender was not significant, indicating that the effect of cooking fuel on LTRI did not vary by gender.

The Durbin-Watson-Hausman test did confirm that stunting was endogenous. The other test results suggested that the instrument (that is, mother’s height) was strong and valid. Further, the Hausman test results indicated that the random-effects approach was appropriate.

## Discussion

LTRI among children is a serious health problem in India. Our findings indicate that the use of solid fuels for cooking has a significant impact on the incidence of LTRI in children under six in India, independent of the child’s age, gender, nutritional status, mother’s education, household wealth and other factors. To the best of our knowledge, this is the first study that uses a longitudinal data to establish the association between IAP and the incidence of LTRI in India. Our findings are consistent with the findings of earlier studies conducted in India [8,10,11,22,23,27].

Stunting in children was not statistically associated with the incidence of LTRI. This finding is not consistent with the findings of earlier studies [8,10,13,18,20,22,32,38]. The results of our study might differ from those of earlier studies because of the issue of endogeneity while estimating the association between stunting and LTRI. None of the earlier studies have treated stunting as endogenous while estimating the association between stunting and LTRI. Moreover, we tried to include severely stunted (HAZ < −3 SD) in place of stunted in the statistical models. But the relationship between severe stunting and LTRI was no different.

Another key finding relates to the presence of other women in the household. Pitt, Rosenzweig [19] argued that the presence of other women in the household is likely to reduce young children’s exposure to solid fuels, thus resulting in lower incidence of LTRI. In our analysis, the presence of other women in the household was not statistically associated with the incidence of LTRI. Similarly, the effect of IAP on LTRI did not vary by gender. Although, in the Indian context, female children are more likely than male children to be exposed to IAP from the use of solid fuels, LTRI are more severe and frequent in male children as a consequence of their narrower peripheral airways [39,40]. Literature on cooking smoke and LTRI also suggests that there are more negative effects on boys than on girls [11].

This study has some limitations. First, we could not measure the exposure levels and patterns of IAP due to the unavailability of such information in YLS. Hence, we could not quantify the relationship between exposure level and risk of LTRI. Second, we were unable to adjust for environmental tobacco smoke as information on tobacco smoking by household members was not collected in the YLS survey. Environmental tobacco smoke is a well-known risk factor of LTRI in young children [41]. Third, our estimate of the effect of IAP on LTRI is likely to be lower than expected due to possible LTRI-related mortality selection. There is every chance of higher LTRI-related mortality in children from households using solid fuels than in children from households using cleaner fuels. However, the impact of this bias is likely to be small, given fewer deaths in the YLS sample. Fourth, our estimate of the impact of IAP on LTRI is also likely to be affected by differential reporting of LTRI by households using solid and cleaner fuels. Differential reporting could happen due to lack of awareness about LTRI. If the households using solid fuels underreport LTRI more than the households using cleaner fuels, then our estimate of the impact of IAP on LTRI is likely to be biased downward. Fifth, there is a possibility that some unobserved time-variant factors might contaminate the relationship between IAP and LTRI. To confirm the absence of such a contamination, we performed a placebo test. We estimated the impact of IAP on the incidence of life threatening diarrhoea (incidence of life threatening diarrhoea is completely unrelated to exposure to smoke). The results suggested no impact of IAP on the incidence of life threatening diarrhoea. This result reassures that our estimates do not capture the indirect effect of other unobserved time-variant risk factors. Sixth, we could not adjust our results for the type of cooking stoves used in the household. This was due to the unavailability of such information in the YLS. Finally, the information on LTRI in YLS is based on the mother’s report. We could not validate the information provided by mothers by cross reference to clinic records or biomarkers. However, in countries where clinical data on LTRI are not available, the symptomatic definition of illness (used here) provides a fairly accurate estimate of LTRI in children under the age of six. Even the Indian DHS uses symptomatic definition of illness to collect information on ARI at the state and national levels [14].

## Conclusions

The findings of our study have important policy implications. About, two-thirds of the Indian population resides in rural areas where the use of solid fuels is still very common. According to the District Level Household Survey – 3 conducted in India in 2007–08 [42], 92% of the rural households were using solid fuels in India. Converting these into numbers will result in a huge population of children who are exposed to IAP from the use of solid fuels for cooking. At the same time, the burden of ARI in children is also tremendously high in India [7]. There is, therefore, an urgent need to make people aware of the health risks associated with the use of solid fuels. The Government of India must promote the use of cleaner fuel and cleaner stoves especially in rural areas.

## Appendix

In the first stage, random effects logistic regression was used to predict stunted growth among children under age six years as a function of mother’s height and other explanatory variables. In the second stage, LTRI was regressed on predicted values of stunting and other variables using the random effects logistic model.

**Second stage equation:**

$$ \begin{array}{l} \log it\left(LTR{I}_{ijkmt}\right)\\ {}\kern9em ={\theta}_{ij}+{\phi}_k+{\varphi}_m+{\delta}_t+{\beta}_1\left(IA{P}_{kmt}\right)+{\beta}_2\left({A}_{ijkmt}\right)+{\beta}_3\left({G}_{ijkmt}\right)\\ {}+{\beta}_4\left({S}_{ijkmt}\right)+{\beta}_5\left({B}_{ijkmt}\right)+{\beta}_6\left({E}_{jkmt}\right)+{\beta}_7\left({W}_{jkmt}\right)+{\beta}_8\left({H}_{kmt}\right)\\ {}+{\beta}_9\left({N}_{kmt}\right)+{\beta}_{10}\left({C}_{kmt}\right)+{\beta}_{11}\left({R}_{kmt}\right)+{\beta}_{12}\left({Q}_{kmt}\right)+{\beta}_{13}\left({I}_{kmt}\right)\\ {}+{\beta}_{14}\left({U}_{kmt}\right)+{\beta}_{15}\left({A}_{kmt}\right)+{\beta}_{16}\left({K}_{kmt}\right)+{\beta}_{17}\left({L}_{kmt}\right)+{\beta}_{18}\left({Z}_{kmt}\right)\\ {}+{\beta}_{18}\left({P}_{mt}\right)+{\mu}_{ijkmt}\end{array} $$

**First stage equation:**

$$ \begin{array}{l} \log it\left({S}_{ijkmt}\right)={\theta}_{ij}+{\phi}_k+{\varphi}_m+{\delta}_t+{\beta}_1\left(M{H}_{jkmt}\right)+{\beta}_2\left({A}_{ijkmt}\right)+{\beta}_3\left({G}_{ijkmt}\right)\\ {}+{\beta}_4\left({S}_{ijkmt}\right)+{\beta}_5\left({B}_{ijkmt}\right)+{\beta}_6\left({E}_{jkmt}\right)+{\beta}_7\left({W}_{jkmt}\right)+{\beta}_8\left({H}_{kmt}\right)\\ {}+{\beta}_9\left({N}_{kmt}\right)+{\beta}_{10}\left({C}_{kmt}\right)+{\beta}_{11}\left({R}_{kmt}\right)+{\beta}_{12}\left({Q}_{kmt}\right)+{\beta}_{13}\left({I}_{kmt}\right)\\ {}+{\beta}_9\left({N}_{kmt}\right)+{\beta}_{10}\left({C}_{kmt}\right)+{\beta}_{11}\left({R}_{kmt}\right)+{\beta}_{12}\left({Q}_{kmt}\right)+{\beta}_{13}\left({I}_{kmt}\right)\\ {}\kern8.5em +{\beta}_9\left({N}_{kmt}\right)+{\beta}_{10}\left({C}_{kmt}\right)+{\beta}_{11}\left({R}_{kmt}\right)+{\beta}_{12}\left({Q}_{kmt}\right)+{\beta}_{13}\left({I}_{kmt}\right)\\ {}\kern8.5em +{\beta}_{14}\left({U}_{kmt}\right)+{\beta}_{15}\left({A}_{kmt}\right)+{\beta}_{16}\left({K}_{kmt}\right)+{\beta}_{17}\left({L}_{kmt}\right)+{\beta}_{18}\left({Z}_{kmt}\right)\\ {}\kern8.5em +{\beta}_{18}\left({P}_{mt}\right)+{\mu}_{ijkmt}\end{array} $$

where *LTRI*_*ijkmt*_ is the health status of the child *i* of the parent *j* in the household with living condition *k* of the community *m* at time *t. θ*_*ij*_ is the child and parent level random effect. *∅*_*k*_*,φ*_*m,*_*δ*_*t*_ are the household, community and year of survey random effects respectively. *IAP*_*kmt*_ is the use of solid cooking fuel in the household *k* of community *m* at time *t. A*_*ijkmt*_,*G*_*ijkmt*_, *S*_*ijkmt*_, and *B*_*ijkmt*_ are the age, gender, nutritional status (stunted) and number of sibling of the index child respectively. E_jkmt_ and W_jkmt_ are the education and working status of mother. Household socio-economic conditions are represented by *H*_*kmt*_ (household head’s education), *N*_*kmt*_(household size), *C*_*kmt*_ (caste), *R*_*kmt*_ (religion), *Q*_*kmt*_ (wealth quintile),*I*_*kmt*_ (income shock/job loss) and *U*_*kmt*_ (rural-urban residence). Household living conditions are represented by *A*_*kmt*_ (other women in the household),*K*_*kmt*_ (wall permeability), *L*_*kmt*_ (roof permeability)and *Z*_*kmt*_ (household crowding). Outdoor air pollution is represented by *P*_*mt*_. Mother’s height *MH*_*jkmt*_ is the instrument for child’s stunting.

The Durbin-Watson-Hausman test was conducted to examine whether stunting was endogenous. Tests were also performed to examine whether the instrument was strong and valid. The Hausman test was also performed to decide between fixed and random effects. The Hausman test basically tests whether the unique errors (*u*_*i*_) are correlated with the regressors Greene 2012) [43].
